# Impact of clinical sample processing time on the performance and T-cell response of the T-SPOT.TB assay

**DOI:** 10.3389/fcimb.2026.1912652

**Published:** 2026-09-10

**Authors:** Yue Chang, Guiren Ruan, Yaxu Liu, Baotong Zhou, Lifan Zhang, Yang Han

**Affiliations:** 1 Department of Infectious Diseases, State Key Laboratory of Complex Severe and Rare Disease, Peking Union Medical College Hospital, Chinese Academy of Medical Sciences and Peking Union Medical College, Beijing, China; 2 Department of Health Care, Peking Union Medical College Hospital, Chinese Academy of Medical Sciences, Beijing, China; 3 Clinical Epidemiology Unit, Peking Union Medical College, International Clinical Epidemiology Network, Beijing, China; 4 Center for Tuberculosis Research, Chinese Academy of Medical Sciences and Peking Union Medical College, Beijing, China

**Keywords:** diagnosis, interferon-gamma release assay, Mycobacterium tuberculosis infection, T-Cell Xtend, T-SPOT.*TB*

## Abstract

**Objectives:**

T-SPOT.*TB*, an interferon-gamma release assay (IGRA) for detecting Mycobacterium tuberculosis infection, requires blood processing within 4 hours, limiting clinical flexibility. T-Cell Xtend™ (TCX) extends permissible storage but is costly. This study evaluated delayed processing impacts on T-SPOT.*TB* consistency in a real-world setting.

**Methods:**

Outpatients undergoing T-SPOT.*TB* testing were prospectively enrolled. Samples were processed at standard timing (≤4 h), after 24-h delay with TCX, and after 24-h delay without TCX. Consecutive-day fresh samples served as within-subject reproducibility references.

**Results:**

Among 443 patients, 282 underwent 24-h delayed testing. Agreement with standard-timing results was 93.3% without TCX (κ=0.859) and 93.0% with TCX (κ=0.860), comparable to fresh-sample reproducibility (95.0%; κ=0.840; all p<0.001). Multivariable analysis identified initial borderline results (OR = 15.21, p<0.001) and 24-h delay (p<0.05) as independent predictors of T-SPOT.TB reversion, regardless of TCX use. Antigen-well spot-forming cell (SFC) counts were significantly reduced after 24-h delay (p<0.001); TCX did not mitigate this decline (p=1.000). Positive-control SFC counts remained stable (p=0.602).

**Conclusions:**

A 24-h processing delay demonstrated high concordance with standard-timing T-SPOT.*TB* results, supporting clinical feasibility. Nevertheless, borderline results and delayed processing were independently associated with result discordance, warranting cautious clinical interpretation.

## 1 Introduction

In 2024, the global count of new tuberculosis (TB) cases reached approximately 10.7 million, with an estimated incidence rate of 131 per 100,000 people. China ranked the fourth among the 30 countries with high TB burden, accounting for 6.5% of the global incidence. There remains a significant gap from the targets set by the “End TB Strategy” for 2025 (World Health Organization, 2024). Latent tuberculosis infection (LTBI) plays a key role in the emergence of new TB cases (Shao et al., 2021). Consequently, screening for LTBI and implementing preventive tuberculosis treatment (TPT) are critical strategies in TB control (World Health Organization, 2024).

T-SPOT.*TB*, a type of interferon-gamma release assay (IGRA) based on the enzyme-linked immunospot (ELISpot) method, is approved by WHO for detecting Mycobacterium tuberculosis (Mtb) infections. It quantifies the number of interferon-gamma (IFN-γ) producing T cells stimulated by exposure to Mtb antigens, specifically ESAT-6 and CFP-10, which manifest as spots (Feng et al., 2021). During T-SPOT.*TB* detection, peripheral blood mononuclear cells (PBMCs) need to be isolated from blood sample (Wang et al., 2012). Due to the rapid decline in cellular function and the diminished capacity for antigen response, cell-mediated immunological methods require the use of blood that is as fresh as possible for accurate analysis (Li et al., 2023). According to the reagent manufacturer’s instructions, testing procedures must be performed within 4 hours of blood collection. This requirement limits the laboratory’s ability to process multiple samples in batches, thus affecting the efficient use of technicians’ time, equipment, and reagents (Wang et al., 2010). In some institutions with inadequate facilities—especially when external sample testing is involved—the distance between the blood collection site and the testing laboratory may impede the timely transportation of samples for same-day processing.

To provide greater flexibility in processing, the manufacturer of T-SPOT.TB has developed a proprietary reagent called T-Cell Xtend™ (TCX). This reagent reportedly allows whole blood samples stored at 10-25 °C to be processed within a 32-hour window without compromising the accuracy of the assay (Talbot et al., 2012; Li et al., 2017). However, TCX’s higher cost limits its widespread use in routine clinical applications. To enhance workflow and efficiency from a clinical application perspective, we processed samples at various intervals post-receipt and compared them with and without TCX. This study aims to investigate the effects of delayed sample processing on detection accuracy in a clinical setting and to assess the performance and feasibility of TCX in diagnostic environments.

## 2 Materials and methods

### 2.1 Study design and participants

We included 443 participants (217 men and 226 women) referred for routine T-SPOT.*TB* testing at the outpatient clinic and divided them into two study arms. The prospective 24-h Delayed Testing Arm included 282 participants enrolled between March and October 2024. Each participant provided a single venous blood sample, which was divided into aliquots for testing under different processing conditions. All 282 samples were tested routinely within 4 h of collection and again after a 24-h delay at room temperature without TCX; 214 of these samples were additionally tested after the same 24-h delay with TCX. Thus, participants in Groups 1–3 required neither repeat venipuncture nor an additional clinic visit.

The Fresh Sample Testing Arm comprised an independent historical repeat-testing reference cohort of 161 patients who underwent T-SPOT.*TB* testing on two consecutive days during routine clinical care between December 2012 and March 2024, with each newly collected blood sample processed within 4 h. These paired results were used to characterize the assay’s baseline short-term variability independent of processing delay.

**Figure 1:**
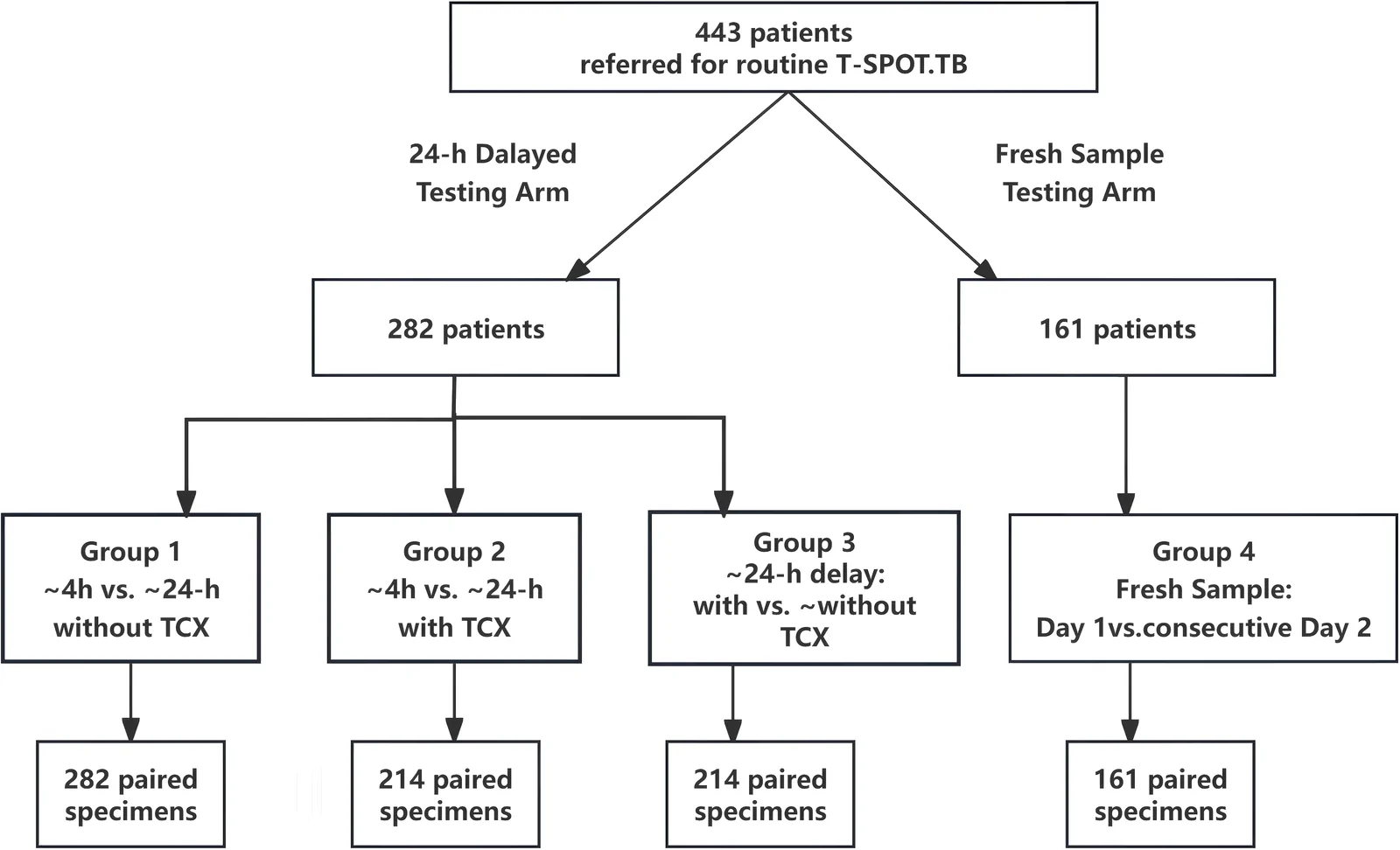
Flow diagram of study participants. A total of 443 participants were included in two study arms. The prospective delayed-processing arm comprised 282 outpatients enrolled from March to October 2024. Samples from the same blood collection were processed under standard conditions within 4 h and again after a 24-h delay without TCX (n = 282). Among these participants, 214 also had sufficient sample available for parallel 24-h processing with TCX, allowing direct paired comparison between the two delayed conditions. The independent historical reproducibility reference cohort comprised 161 outpatients who underwent clinically indicated T-SPOT.TB testing on two consecutive days between 2012 and 2024, with a newly collected blood sample processed within 4 h on each day. Numbers shown in the diagram indicate the participants included in each paired comparison.

The study flow is shown in **Figure 1**. The study was approved by the Ethics Committee of Peking Union Medical College Hospital (No. K5645) and conducted in accordance with the Declaration of Helsinki.

### 2.2 T-SPOT.*TB*

Blood samples were collected in lithium heparin tubes and transported at room temperature. To minimize bias, laboratory technicians were blinded to the clinical information of the samples. Routine clinical testing was performed within 4 hours of sample collection. For blood samples subjected to 24-hour delayed testing, after storage at room temperature for 24 hours, they were divided into two aliquots, which were tested with or without the addition of TCX, respectively. The T-SPOT.*TB* test, the use of TCX, and the interpretation of test results all strictly followed the manufacturer’s standard protocols. Spot-forming cells (SFCs) were counted using an automated AID ELISPOT reader (AID Autoimmun Diagnostika GmbH), and test results were recorded according to the approved interpretation algorithm: a result was defined as “reactive” if the SFC count in either antigen well exceeded that in the nil control by at least 6 (if the SFC count in the nil control was 0∼5) or was at least double the SFC count in the nil control (if SFC count in nil control was 6∼10). If the above criteria were not met and the SFC count in the positive control was normal, the test result was defined as “non-reactive”. Results where the highest SFC count from either antigen well showed a difference of 5, 6, or 7 spots compared to the Nil control were defined as “borderline”. In T-SPOT.*TB* testing, “conversion” is defined as a shift from non-reactive to reactive upon retesting, while “reversion” refers to a shift from reactive to non-reactive.

### 2.3 Statistical analysis

Continuous data with normal distribution were expressed as mean ± standard deviation (SD), and paired t-tests were used for intrapatient comparisons of serial samples; data without normal distribution were described as median and interquartile range (IQR), with the Wilcoxon signed-rank test applied for paired intrapatient analyses. Categorical variables were compared using the chi-square test. Paired qualitative classifications were compared using the exact McNemar test, whereas agreement was quantified using Cohen’s κ with 95% confidence intervals. Univariate and multivariable logistic regression models were performed to identify factors influencing result discrepancies. Variables considered clinically relevant or those that showed a significant association with result discrepancies in univariate analysis were entered into the multivariable logistic regression model (backward LR, entry threshold: P < 0.05, removal threshold: P > 0.1). Statistical analyses were performed with SPSS software (SPSS^®^ for Windows™, version 21.0, SPSS Inc., Chicago, IL, USA), GraphPad Prism software (GraphPad Software^®^ for Windows™, version 9.0, USA). and R Statistical Software (version 4.2.2).

## 3 Results

### 3.1 The characteristics of study population

A total of 443 patients referred for T-SPOT.TB testing were enrolled in this study, of whom 49.0% were male, with a mean age of 48 ± 17 years. Among all participants, 32.5% met the diagnostic criteria for immune-mediated inflammatory diseases (IMID) (Schett et al., 2021). A total of 1100 valid specimens were included for outcome analysis (including 282 standard-timing specimens, 282 24-h-delayed specimens without TCX, 214 24-h-delayed specimens with TCX, and 322 specimens from the historical reproducibility reference). The baseline characteristics of the participants are shown in **Table 1**.

**Table 1:** General characteristics of study participants.

Characteristic	Delayed testing arm (n=282)	Fresh sample testing arm (n=161)
Age, yr, mean ± SD	48 ± 15	48 ± 19
Male, n (%)	139 (49.3)	78 (48.4)
Clinical disease categories, n (%)		
Immune and Connective Tissue Disorders	104 (36.9)	30 (18.6)
Cardiovascular and Respiratory Disorders	30 (10.6)	26 (16.1)
Digestive, Renal, and Metabolic Disorders	35 (12.4)	19 (11.8)
Infectious Diseases	20 (7.1)	45 (28.0)
Oncologic Diseases	7 (2.5)	5 (3.1)
Unspecified and Other Conditions	86 (30.5)	36 (22.4)
IMID, n (%)	111 (39.4)	33 (20.5)

^a^IMID, Immune-Mediated Inflammatory Diseases.

### 3.2 Evaluation of consistency in T-SPOT.*TB* results with different clinical processing times

**Figure 2:**
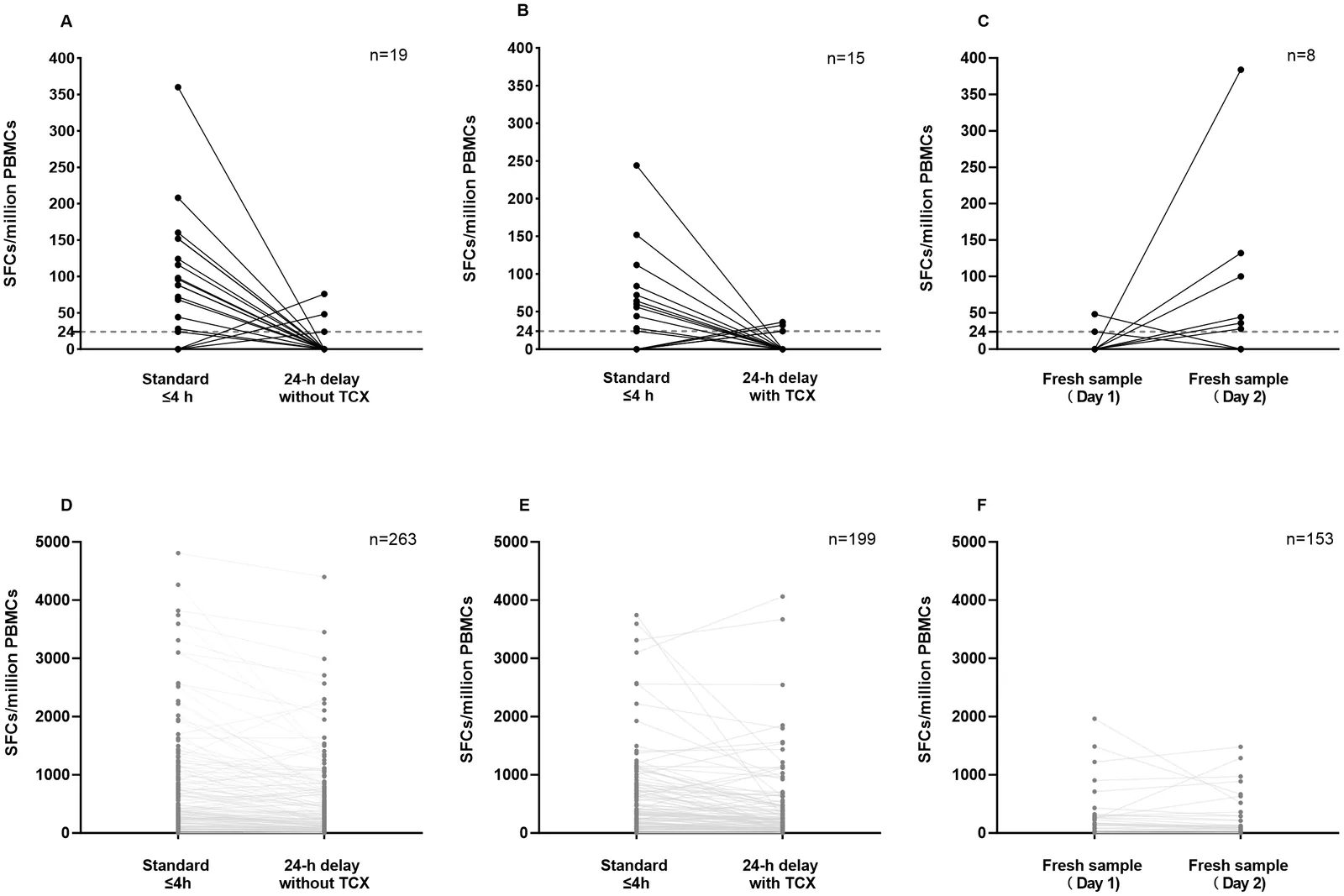
Paired T-SPOT.TB response patterns with concordant and discordant outcomes during different processing. **(A–C)** show discordant pairs for standard ≤4-h processing versus 24-h delay without TCX **(A)**, standard ≤4-h processing versus 24-h delay with TCX **(B)**, and fresh sample Day 1 versus Day 2 **(C)**. **(D–F)** show the corresponding concordant pairs for the same three comparisons. Each line represents one participant. The figure is intended as a descriptive visualization of paired response patterns.

In the 24-h delayed testing arm, 282 patients underwent standard timely T-SPOT.TB testing, resulting in 178 reactive results (63.1%) and 104 non-reactive results (36.9%). When these samples were retested after a 24-h delay without the addition of TCX, 19 cases showed inconsistent results, with 9.0% (16/178) reverting from reactive to non-reactive and 2.9% (3/104) converting from non-reactive to reactive (**Figure 2A**). The agreement between timely testing and 24-h delayed testing without TCX was 93.3% (kappa=0.859, p<0.001) (**Table 2A**). To further evaluate the impact of TCX on delayed processing, 214 samples were retested with TCX after a 24-h delay. Among these, 10.0% (11/110) reverted from reactive to non-reactive and 3.8% (4/104) converted from non-reactive to reactive (**Figure 2B**), with an agreement of 93.0% (kappa=0.860, p<0.001) compared with the standard timely testing results (**Table 2B**).

**Table 2:** Agreement between serial T-SPOT.*TB* results with different clinical processing times.

A. Standard ≤4-h processing vs 24-h delay without TCX (n=282)			
	24-h delay without TCX		
Standard ≤4-h result	Reactive	Non-reactive	Total
Reactive	162 (91.0)*Concordant*	16 (9.0)*Reversion*	178
Non-reactive	3 (2.9)*Conversion*	101 (97.1)*Concordant*	104
Total	165	117	282
Overall agreement, 93.3%; Cohen κ=0.859 (95% CI, 0.798–0.920); exact McNemar P = 0.0044.			

B. Standard ≤4-h processing vs 24-h delay with TCX (n=214)			
	24-h delay with TCX		
Standard ≤4-h result	Reactive	Non-reactive	Total
Reactive	99 (90.0) *Concordant*	11 (10.0) *Reversion*	110
Non-reactive	4 (3.8) *Conversion*	100 (96.2) *Concordant*	104
Total	103	111	214
Overall agreement, 93.0%; Cohen κ=0.860 (95% CI, 0.792–0.928); exact McNemar P = 0.1185.			

C. Fresh Day 1 vs consecutive Day 2 testing (n=161)			
	Fresh sample (consecutive Day 2)		
Fresh sample (Day 1)	Reactive	Non-reactive	Total
Reactive	27 (93.1) *Concordant*	2 (6.9) *Reversion*	29
Non-reactive	6 (4.5)*Conversion*	126 (95.5)*Concordant*	132
Total	33	128	161
Overall agreement, 95.0%; Cohen κ=0.840 (95% CI, 0.733–0.947); exact McNemar P = 0.2891.			

Data are presented as n (%) except for totals. “Concordant” denotes the same qualitative classification in paired tests; “reversion” denotes reactive to non-reactive change; and “conversion” denotes non-reactive to reactive change. Cohen’s κ quantifies agreement beyond chance, whereas the exact McNemar test was used to assess differences in paired reactive/non-reactive proportions. All P values are two-sided. TCX, T-Cell Xtend™.

In the fresh sample testing arm, 29 of 161 patients (18.0%) tested reactive on the first day. Of these patients, 6.9% (2/29) reverted to non-reactive on the following day, whereas 4.5% (6/132) of initially non-reactive patients converted to reactive (**Figure 2C**). Overall concordance between the two consecutive-day fresh-sample results was 95.0% (kappa=0.840, p<0.001) (**Table 2C**).

Among the corresponding concordant pairs, within-subject variation in SFC magnitude was observed without a change in qualitative T-SPOT.TB classification across the three comparison settings (**Figures 2D–F**), providing a direct contrast to the discordant trajectories shown in **Figures 2A–C**.

### 3.3 Impact factors of the diagnostic inconsistency of T-SPOT.*TB* test

We analyzed the potential factors associated with inconsistent T-SPOT.TB results (reversion or conversion) across different processing times. Multivariable logistic regression showed that an initial borderline T-SPOT.*TB* result (OR = 15.21, 95% CI: 4.52–51.19, P<0.001) and 24-h delayed sample processing (regardless of TCX) were independent factors of T-SPOT.*TB* reversion. Furthermore, a borderline result in the 24-h delayed T-SPOT.*TB* test (OR = 23.12, 95% CI: 5.59–95.70, P<0.001) emerged as an independent factor of T-SPOT.*TB* conversion (**Table 3**).

**Table 3:** Factors associated with inconsistent results of T-SPOT.*TB* under different processing times.

Variable	Reversion				Conversion			
	Univariate analysis		Multivariable analysis		Univariate analysis		Multivariable analysis	
	OR (95%CI)	P value	OR (95%CI)	P value	OR (95%CI)	P value	OR (95%CI)	P value
Gender								
Male	1				1			
Female	1.05 (0.50-2.22)	0.890			0.84 (0.28-2.54)	0.762		
Age	1.02 (0.99-1.04)	0.133	1.02 (0.99-1.05)	0.094	0.98 (0.95-1.01)	0.191		
IMID status								
No	1				1			
Yes	0.79 (0.35-1.77)	0.568			0.78 (0.24-2.57)	0.684		
Borderline Results in Initial T-SPOT.TB Test								
No	1				1			
Yes	14.03 (4.37-45.06)	**<0.001**	15.21 (4.52-51.19)	**<0.001**	5.61 (0.66-47.86)	0.115		
Borderline Results in Repeated T-SPOT.TB Test								
No	1				1		1	
Yes	2.20 (0.49-9.91)	0.303			13.22 (3.75-46.58)	**<0.001**	23.12 (5.59-95.70)	**<0.001**
Group								
Fresh sample testing (on consecutive days)	1				1		1	
24-h delay testing. (without TCX)	4.65 (1.06-20.52)	**0.042**	5.28 (1.14-24.52)	**0.034**	0.29 (0.07-1.18)	0.084	0.15 (0.03-0.74)	**0.020**
24-h delay testing. (with TCX)	4.23 (0.92-19.36)	0.063	5.03 (1.04-24.33)	**0.044**	0.51 (0.14-1.85)	0.307	0.40 (0.10-1.54)	0.180

Multivariable logistic regression model included gender, age, IMID status, borderline results in initial T-SPOT.TB test, borderline results in repeated T-SPOT.*TB* test, and group.

Bold values indicate statistically significant results (P < 0.05).

### 3.4 Evaluation of immune response intensity of T-SPOT.TB results under different clinical processing times

**Figure 3:**
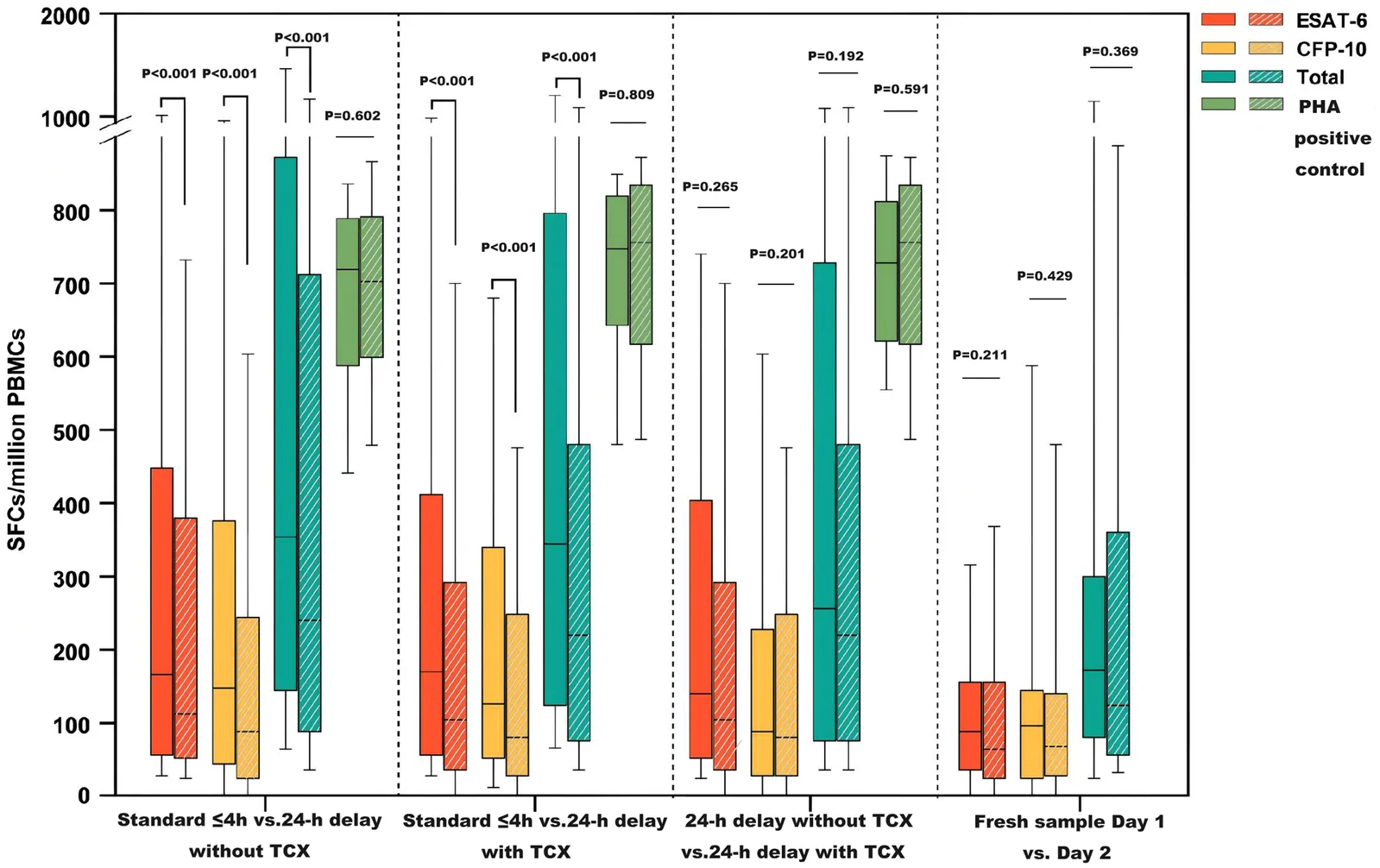
Comparison of SFC counts under different sample processing timelines. The box plots show SFC counts for ESAT-6, CFP-10, total antigens, and positive results across four comparison groups: standard ≤4-h vs. 24-h delay without TCX, standard ≤4-h vs. 24-h delay with TCX, 24-h delay without vs. with TCX, and the fresh sample retesting group. The central line in each box represents the median, the box represents the interquartile range (IQR), and the whiskers extend to the minimum and maximum values. Statistical comparisons were performed using the Wilcoxon signed-rank test with exact P value.

Additionally, to evaluate the impact of delayed processing on the intensity of the antigen-specific immune response, we compared SFC frequencies in patients with initially positive T-SPOT.*TB* results. For samples retested after a 24-h delay, antigen-specific SFC counts were significantly lower than those obtained with standard ≤4-h processing, both without TCX (ESAT-6, P < 0.001; CFP-10, P < 0.001; Total, P < 0.001) and with TCX (ESAT-6, P < 0.001; CFP-10, P < 0.001; Total, P < 0.001). No significant differences in antigen-specific SFC counts were observed between the two 24-h delayed-processing conditions (ESAT-6, P = 0.265; CFP-10, P = 0.201; Total, P = 0.192). PHA positive-control responses remained stable across the three processing conditions (P = 0.602). In the consecutive-day fresh-sample group, antigen-specific SFC counts also showed no significant differences between Day 1 and Day 2 (ESAT-6, P = 0.211; CFP-10, P = 0.429; Total, P = 0.369, respectively) (**Figure 3**).

## 4 Discussion

A 24-h delay in T-SPOT.TB processing, with or without TCX, largely preserved qualitative concordance with standard 4-h processing, even though antigen-specific IFN-γ responses were reduced. The divergence between largely preserved qualitative concordance and attenuated quantitative immune responses has important clinical implications, suggesting that delayed processing may offer greater flexibility in sample handling without substantially compromising overall test classification, while warranting particular caution when interpreting results close to the diagnostic threshold.

The purpose of adding TCX to blood samples prior to sample processing is to mitigate the effects of delayed processing, which typically leads to a quantitative reduction in IFN-γ-secreting T cells (Pai et al., 2014; Banaei et al., 2016). T-Cell Xtend™ is a patented antibody reagent designed to cross-link red blood cells with granulocytes, effectively preventing unwanted granulocyte accumulation in the peripheral blood mononuclear cell (PBMC) layer of long-stored blood samples. Theoretically, this stabilization could help preserve assay performance by improving PBMC separation purity in stored samples, potentially yielding readouts comparable to those obtained with samples processed within the standard 4-h window (Mazurek et al., 2010). Although manufacture claims a consistency rate of 96.6% (340/352) for T-SPOT results, with or without TCX, only a few published studies have investigated TCX’s impact on alleviating the effects of delayed processing on T-SPOT.*TB.*

Our findings demonstrated that the diagnostic agreement between 24-hour delayed processing—both with and without TCX—and standard-timing testing was substantial, with kappa values of 0.860 and 0.859, respectively. In addition, the concordance between results from fresh samples tested on two consecutive days was 95.0% (kappa=0.840). The high qualitative concordance observed in our study is consistent with previous evaluations of delayed T-SPOT.TB processing. Lenders et al. reported 97.1% agreement between immediate and 24-h delayed processing without TCX (Lenders et al., 2010), while Wang et al. reported 98.2% overall agreement, although the corresponding kappa value was lower (κ = 0.493), likely reflecting the low prevalence of positive results in that study population (Wang et al., 2010). Small studies evaluating TCX have likewise reported preserved qualitative agreement with processing delays of up to 33 h (Bouwman et al., 2012; Wang et al., 2012). Together, these findings support a degree of flexibility in sample-processing time. Despite this high overall concordance, reversions from reactive to non-reactive results were more frequent than conversions in the delayed-processing groups (9.0% and 9.9% vs. 2.9% and 3.8%, respectively). Together with the observed reduction in antigen-stimulated SFC counts after delayed processing, this asymmetric pattern supports a consistent shift toward attenuated antigen-specific T-cell responses during prolonged storage, although the precise mechanisms driving individual changes in qualitative classification remain to be established.

Borderline results and delayed processing were the principal factors associated with T-SPOT.TB discordance, rather than demographic characteristics or underlying comorbidities. An initial borderline result was strongly associated with reversion (OR = 15.21, P < 0.001), whereas a borderline result at repeat testing was independently associated with conversion (OR = 23.12, P < 0.001). This bidirectional instability is consistent with other evidence that threshold-proximal IGRA results are particularly susceptible to categorical change on repeat testing. A recent systematic review and meta-analysis demonstrated increased conversion and reversion among QFT results within predefined borderline ranges (Wang et al., 2025), while Ringshausen et al. documented substantial short-term variability in T-SPOT.TB responses, with regression toward the mean contributing to an approximately 24% decline in mean IFN-γ responses between successive measurements (Ringshausen et al., 2011). We therefore hypothesize that delayed processing may further predispose threshold-proximal samples to reversion by attenuating detectable antigen-specific SFC responses. This mechanism remains speculative, as regression toward the mean and other sources of assay variability cannot be excluded. Given this inherent instability around the diagnostic threshold, repeat testing may be considered for patients with an initial borderline result to reduce the potential for misinterpretation.

We also analyzed the intensity of the antigen-specific immune response, which directly reflects the functional integrity of T cells under various processing conditions. Moreover, the stability of SFCs across consecutive two days for fresh samples (p > 0.05) underscores the robust reproducibility of the T-SPOT.TB when conducted within the standard 4-h window. Notably, TCX failed to attenuate the reduction in antigen-specific SFC responses observed after 24 h of storage. One possible explanation is that the principal effect of TCX—reducing granulocyte carryover during PBMC isolation—may not address the time-dependent functional changes that occur in antigen-responsive T cells during prolonged ex vivo storage. The preservation of mitogen-induced responses in parallel with diminished antigen-specific SFC counts further suggests that this phenomenon may reflect selective impairment of antigen-dependent cellular responses rather than generalized loss of T-cell functionality. Such attenuation could plausibly arise from progressive deterioration in antigen-presenting cell function, reduced T-cell receptor signaling capacity, or preferential loss of antigen-responsive effector and effector-memory T-cell populations during storage. This mechanistic explanation remains a hypothesis and would benefit from confirmation using flow-cytometric assessment of T-cell viability and functional phenotype across the storage interval in future studies. In clinical practice, when considering the delayed processing of samples, it is essential to fully account for the potential reduction in SFCs. Additionally, the use of TCX should be weighed comprehensively against factors such as cost and testing requirements to avoid its indiscriminate application.

This study has several limitations. First, the immunological stability of T cells in outpatients may differ from that of inpatients, whose PBMCs might be more fragile due to acute illness, systemic inflammation, or immunosuppressive therapy. Second, no specific exclusion criteria were applied, so residual confounding from underlying immune status cannot be fully excluded, this design was nonetheless intended to better reflect real-world clinical practice and improve the representativeness of our cohort. Third, although a significant subset of participants had IMID, our analysis focused on diagnostic categories without accounting for specific medication histories. This lack of detailed data on immunosuppressive therapy may introduce residual confounding. Fourth, the within-subject reproducibility reference was derived from a historical cohort (2012–2024), as collecting samples on consecutive days specifically for research was constrained by clinical ethics. While the long timeframe could theoretically introduce variability, the T-SPOT.TB assay maintained high stability throughout this period due to consistent laboratory protocols and standard reagents. Although the proportion of patients with IMID varied slightly over time, multivariable analysis confirmed that IMID status was not a significant confounder for result inconsistency. Finally, processing delays beyond 24 h were not evaluated because the present study focused on a clinically relevant delay interval. Accordingly, our findings cannot define the maximum acceptable processing window for T-SPOT.TB testing, either with or without TCX.

## 5 Conclusion

In summary, our study provides comparable data on blood sample results between standard timely testing and delayed processing. Samples processed after a 24-hour delay, regardless of the addition of TCX, showed high consistency with those processed promptly. However, there was a significant reduction in SFCs in antigen wells, and the addition of TCX did not mitigate this decline. The presence of borderline results in standard timely testing was identified as an independent risk factor for result inconsistency, with sample processing delays further exacerbating the risk of reversion. The findings suggest that while 24-h delayed processing of T-SPOT.*TB* samples is clinically feasible to some extent, both borderline results and delayed protocols should be interpreted and selected cautiously within the clinical context.

## Data Availability

The original contributions presented in the study are included in the article/supplementary material. Further inquiries can be directed to the corresponding authors.
